# AI-driven intelligent training enhances clinical competence in oncology residency: a randomized controlled trial

**DOI:** 10.3389/fmed.2026.1768388

**Published:** 2026-03-12

**Authors:** Fei Ji, Weikai Xiao, Xi Li

**Affiliations:** 1 Department of Breast Cancer, Cancer Center, Guangdong Provincial People's Hospital (Guangdong Academy of Medical Sciences), Southern Medical University, Guangzhou, China; 2 Department of Breast Cancer, Guangzhou Red Cross Hospital, Guangzhou, China; 3 Department of Obstetrics and Gynecology, Guangzhou First People's Hospital, Guangzhou, China

**Keywords:** artificial intelligence, learning analytics, medical education, mixed reality, oncology, residency training

## Abstract

**Background:**

Rapid advances in artificial intelligence (AI) offer new opportunities to address persistent challenges in healthcare professions education, particularly in oncology residency training, where rapidly evolving knowledge, complex decision-making, and limited high-fidelity practice environments hinder competency development. However, evidence from rigorously evaluated educational interventions remains limited.

**Methods:**

We conducted a randomized controlled trial involving 124 breast oncology residents from three tertiary hospitals. Participants were randomly assigned to an AI-empowered intelligent teaching (AIEIT) group (*n* = 62) or a control group receiving conventional training (*n* = 62). The AIEIT model integrated a dynamic knowledge graph for personalized learning, a virtual patient–AI mentor system for adaptive skills training, a mixed-reality multidisciplinary team platform for collaborative decision-making, and a learning analytics dashboard for continuous feedback. Outcomes included knowledge acquisition, clinical reasoning, procedural competence, collaborative performance, cognitive efficiency, and longitudinal clinical outcomes.

**Results:**

The AIEIT group outperformed the control group across all domains, demonstrating superior mastery of theoretical knowledge, higher procedural accuracy, and greater multidisciplinary collaboration (*all P* < 0.001). Cognitive workload and training time were significantly reduced, while technology adaptability and evidence-based practice utilization markedly improved. At 3-month follow-up, the AIEIT group maintained higher knowledge retention (91.2 ± 3.5% vs 76.8 ± 8.4%, *P* < 0.001) and better clinical outcomes, including fewer postoperative complications and higher patient satisfaction.

**Conclusions:**

This study demonstrates that an AI-driven, closed-loop educational model can substantially enhance clinical competence formation in oncology residency training. By integrating data-driven personalization, human–AI collaboration, and virtual–real learning environments, the AIEIT framework offers a scalable and evidence-based approach for advancing healthcare professions education.

## 1 Introduction

The rapid advancement of artificial intelligence (AI), big data analytics, and virtual simulation technologies has profoundly reshaped the landscape of medical education (1–5). Within this transformation, the paradigm of intelligent education emphasizes adaptive learning pathways, personalized feedback mechanisms, and data-driven instructional design (6–8). This evolution signifies a shift from traditional knowledge transmission toward competency-based medical education, which prioritizes analytical thinking, clinical reasoning, and interdisciplinary collaboration (9–12).

Breast oncology exemplifies a knowledge-intensive and rapidly evolving specialty, characterized by continual innovations in molecular subtyping, genomic profiling, and multimodal treatment strategies (13–15). Conventional didactic instruction often fails to deliver timely, personalized updates aligned with rapidly changing clinical guidelines, while the complexity of diagnostic workflows and multidisciplinary team (MDT) decision-making limits opportunities for structured clinical reasoning training and authentic team-based practice (16, 17). These challenges are particularly pronounced in standardized residency training, where static curricula, limited exposure to complex decision-making scenarios, and delayed or inconsistent feedback mechanisms hinder the systematic development of clinical competence (18–20).

To address these deficiencies, this study introduces an AI-empowered intelligent teaching (AIEIT) model grounded in the principles of data-driven instruction, human–machine collaboration, and virtual–real integration. Specifically, the dynamic AI-based knowledge graph was designed to address knowledge obsolescence by continuously aligning learning content with up-to-date clinical guidelines and individual learning gaps; the mixed-reality (MR)–enhanced virtual MDT platform targets deficiencies in interdisciplinary collaboration and clinical reasoning by enabling immersive, case-based decision-making; and the virtual patient–AI mentor system directly responds to limited hands-on opportunities by providing risk-free, feedback-rich procedural and reasoning practice. Supported by a learning analytics–based evaluation framework, which compensates for inconsistent assessment in traditional training by enabling continuous competency tracking and targeted intervention, this comprehensive approach aims to overcome persistent limitations in conventional medical education. Through empirical validation, this study demonstrates how the AIEIT paradigm enhances residents' clinical reasoning, professional competence, and collaborative capability, thereby linking specific educational challenges to corresponding system features and measurable training outcomes, and providing a scalable framework for medical education reform in the era of digital intelligence.

## 2 Materials and methods

### 2.1 Study design and ethical considerations

A randomized controlled trial was conducted involving 124 resident physicians specializing in breast oncology from three urban, tertiary, teaching hospitals located in the same metropolitan region, all of which are nationally accredited centers for standardized residency training in breast oncology. All participating hospitals followed a unified residency curriculum and identical clinical training requirements, minimizing inter-institutional variability. Participants were recruited using a consecutive sampling strategy during the study period and were randomly assigned at the individual level to either the AI-empowered intelligent teaching (AIEIT) group (*n* = 62) or the control group (*n* = 62). Randomization was performed using a computer-generated random sequence without stratification by center, as baseline training structures, resident characteristics, and educational requirements were comparable across the participating institutions.

The AIEIT group received the AI-empowered intelligent teaching intervention, whereas the control group received standard residency training, consisting of conventional didactic lectures, guideline-based case discussions, supervised clinical practice, and periodic formative assessments, without access to AI-driven personalization, virtual patient systems, mixed-reality (MR)–based multidisciplinary team (MDT) simulations, or learning analytics dashboards. The intervention spanned an entire 3-month full-time clinical rotation in breast oncology.

Inclusion criteria were: (1) enrollment in an accredited breast oncology residency rotation; (2) completion of core general oncology training; and (3) no prior exposure to AI-assisted, virtual patient–based, or mixed-reality educational systems. Exclusion criteria included prior participation in AI-based medical education programs, concurrent enrollment in other educational intervention studies, or incomplete participation in the training rotation.

Participants were randomized 1:1 to the AIEIT or control group using a computer-generated random sequence prepared by an independent statistician. Allocation concealment was ensured through sequentially numbered, opaque, sealed envelopes. Due to the nature of the educational intervention, participants and instructors were not blinded. However, outcome assessors for subjective measures, including clinical reasoning, case presentation, and teamwork, were blinded to group allocation and were not involved in intervention delivery. Objective outcomes were collected or scored using predefined criteria.

The study protocol was reviewed and approved by the Institutional Review Boards of all participating hospitals. Written informed consent was obtained from all participants prior to enrollment, and all procedures adhered to ethical standards for confidentiality and data protection.

### 2.2 AI-empowered intelligent teaching (AIEIT) framework

**Figure 1:**
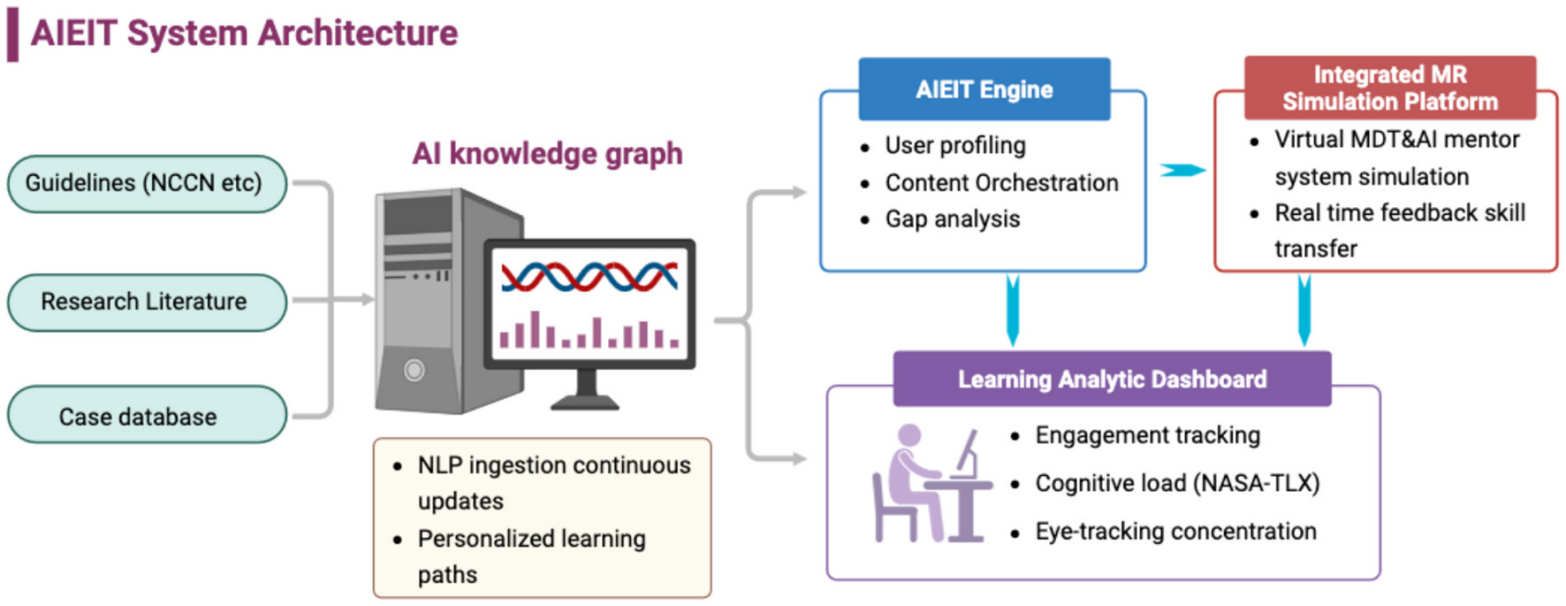
AI-empowered intelligent teaching (AIEIT) sytem architecture. Created with BioRender.com.

The AIEIT model was built upon our proposed framework comprising four core components (Figure 1):

#### 2.2.1 AI-Powered knowledge graph

We developed a dynamic AI-powered knowledge graph using natural language processing (NLP) to extract and structure key information from up-to-date clinical guidelines (including NCCN), peer-reviewed literature, and de-identified educational case materials curated by senior breast oncology specialists. All sources were manually reviewed and validated to ensure educational accuracy and clinical relevance.

The knowledge graph was updated monthly to incorporate newly released guidelines and high-impact studies. It supported three core functions: (1) automated knowledge updating, (2) performance-adaptive learning path generation, and (3) competency gap–driven content recommendation. Personalization was implemented through rule-based and performance-adaptive logic, adjusting learning modules based on prior assessment results, task accuracy, and completion patterns.

Adaptive algorithms were trained exclusively on aggregated and anonymized learner–content interaction data generated during pilot educational use, without access to real-world patient data or electronic health records. The system functioned solely as an educational support tool and did not generate autonomous diagnostic or therapeutic recommendations. Faculty members retained full oversight of content delivery, feedback, and learning objectives.

#### 2.2.2 Mixed-reality MDT training platform

A mixed-reality (MR) multidisciplinary team (MDT) training platform was developed to provide an immersive, interactive learning environment. The platform incorporated approximately 20 standardized MDT scenarios covering early-stage, locally advanced, and complex breast cancer cases, with progressively increasing clinical and decision-making complexity. Learners participated in real-time collaborative decision-making with virtual MDT members, following workflows aligned with clinical guidelines. All scenarios were predefined, standardized, and reviewed by senior clinicians before deployment. Embedded AI-driven virtual specialists provided structured, evidence-based feedback during training and captured performance metrics related to communication, coordination, and information integration. Participants engaged in MR-based MDT training for 1–2 supervised sessions per week, each lasting 60–90 min. AI components facilitated guided discussion and reflective learning but did not generate autonomous clinical decisions or treatment recommendations.

#### 2.2.3 Virtual patient-AI mentor system

A Virtual Patient–AI Mentor System was developed to support clinical skill training in a risk-free environment. The system included approximately 25 virtual patient cases spanning diagnostic evaluation, treatment planning, and procedural decision-making, with scenario difficulty dynamically adjusted according to learner performance.

Virtual patients exhibited adaptive responses to learner actions, supported by AI-guided procedural prompts and immediate error feedback. The AI mentor was built using de-identified educational cases and expert-curated dialogue templates, with all feedback constrained to predefined competency frameworks. The system did not provide real-world clinical instructions or independent medical advice.

Learner interactions were recorded and summarized to support instructor-facilitated debriefing and reflective review. Faculty members reviewed performance reports and retained responsibility for all educational supervision.

#### 2.2.4 Learning analytics dashboard

A learning analytics dashboard was developed to enable continuous, data-driven evaluation of learner performance. The platform monitored learning behaviors, assessment results, and clinical decision trajectories, generating multidimensional competency profiles across cognitive, technical, and collaborative domains.

Performance trends and competency gaps were visualized using descriptive and inferential analytics, without automated summative judgment or replacement of instructor evaluation. Real-time formative feedback was delivered through interactive visual interfaces to support learner self-assessment and adaptive learning.

Instructors used dashboard analytics during scheduled feedback sessions to guide personalized educational interventions and track longitudinal progress, thereby optimizing targeted educational support and overall training effectiveness.

### 2.3 Assessment methods

A competency-based assessment framework was used to evaluate knowledge acquisition, clinical reasoning, technical skills, collaborative competence, learning efficiency, and long-term outcomes. All assessments were conducted using standardized protocols at predefined time points by faculty members not involved in participants' routine supervision. Unless otherwise specified, assessors were blinded to group allocation.

#### 2.3.1 Knowledge acquisition and clinical reasoning

Knowledge acquisition and clinical reasoning were assessed using standardized theoretical examinations, simulated clinical case analyses, and concept-map evaluation. Written examinations assessed mastery of core and advanced breast oncology concepts, including molecular classification, systemic therapy selection, and guideline-based management. Examination content was developed according to national residency training objectives and reviewed for content validity by three senior breast oncology educators. Clinical reasoning was evaluated through standardized simulated cases. Participants independently formulated diagnostic assessments and treatment plans, which were scored using predefined rubric-based criteria aligned with competency-based education frameworks, focusing on diagnostic accuracy, therapeutic appropriateness, and evidence integration. Concept-map analysis assessed knowledge structure and integration based on the number of nodes (conceptual breadth) and cross-links (integrative reasoning). Evidence-based practice was evaluated by counting high-level references cited per decision task; only peer-reviewed guidelines and high-quality clinical studies were included.

#### 2.3.2 Technical skills and procedural competence

Technical proficiency was assessed through objective performance-based evaluations in standardized simulated settings. Imaging interpretation skills were measured using validated test sets for breast ultrasound BI-RADS classification and MRI evaluation, with accuracy determined against expert consensus standards. Procedural competence was assessed by the success rate and operation time of ultrasound-guided biopsy performed on simulation models. Surgical skills were evaluated using simulated surgical tasks, with margin prediction accuracy and aesthetic outcome scores assessed using predefined scoring criteria. Skill retention and transferability were reassessed 3 months after training using comparable assessment formats to evaluate longitudinal competency maintenance and adaptation to novel scenarios.

#### 2.3.3 Collaborative and professional competence

Collaborative and professional competencies were evaluated during standardized simulated multidisciplinary team (MDT) sessions and structured case discussions. Core competencies—including case presentation, imaging interpretation, and treatment planning—were assessed using rubric-based scoring systems aligned with national residency training standards. Team collaboration was evaluated using structured behavioral checklists assessing communication efficiency, active listening, and spontaneous leadership. Professional literacy was assessed using validated instruments measuring patient communication, ethical decision-making, and burnout incidence. Subjective assessments were independently rated by two senior faculty members blinded to group allocation. Inter-rater reliability was assessed using intraclass correlation coefficients, demonstrating acceptable agreement across subjective measures.

#### 2.3.4 Learning efficiency and long-term outcomes

Learning efficiency was evaluated using behavioral analytics automatically recorded by the AIEIT platform, including weekly study time and personalized content completion rate. Cognitive workload was assessed using the NASA–TLX scale following standardized learning tasks. Technology adaptability was evaluated using validated questionnaires measuring perceived usefulness, ease of use, and digital proficiency. Eye-tracking analysis assessed fixation concentration during predefined tasks and its association with task completion performance. Long-term outcomes were evaluated at 6-month follow-up and included standardized knowledge retention testing, specialty examination performance, and clinical indicators. Clinical outcomes comprised postoperative complication rates, average hospital stay, and patient satisfaction, derived from institutional clinical records under appropriate de-identification. Innovation capability and continuing education participation were additionally assessed using structured questionnaires.

### 2.4 Data analysis

Sample size was calculated *a priori* based on an expected moderate-to-large effect size (Cohen's d ≈ 0.6) from prior educational studies, with a two-sided α of 0.05 and 80% power, yielding a required sample of 58 participants per group. To allow for attrition, 124 residents were enrolled. Quantitative analyses were performed using SPSS 26.0 and R. Continuous variables are presented as mean ± standard deviation and compared using independent *t*-tests or repeated-measures ANOVA, as appropriate. Binary outcomes were analyzed using χ^2^ tests. Qualitative learning analytics data were examined using thematic analysis. Statistical significance was defined as *P* < 0.05 (two-sided). The primary outcome was knowledge acquisition and clinical reasoning performance, assessed by standardized examinations and simulated clinical cases at the end of training. Secondary outcomes included technical skills, collaborative competence, learning efficiency, and cognitive workload, while long-term knowledge retention and clinical indicators were considered exploratory outcomes. Given the exploratory nature of this educational randomized controlled trial, no formal adjustment for multiple comparisons was applied; results were interpreted within a predefined hierarchical framework and alongside effect size estimates.

## 3 Results

### 3.1 Participant characteristics

A total of 124 residents were enrolled and randomized equally into the AIEIT and control groups. Baseline demographic and training characteristics were comparable between groups, with no statistically significant differences observed (Table 1).

**Table 1:** Baseline characteristics of participants.

**Characteristic**	**AIEIT group (*n* = 62)**	**Control group (*n* = 62)**	***P* value**
Age (years), mean ± SD	27.8 ± 2.1	28.1 ± 2.3	0.46
**Sex**, ***n*** **(%)**			
Female	48 (77.4%)	46 (74.2%)	0.68
Male	14 (22.6%)	16 (25.8%)	
**Postgraduate year**, ***n*** **(%)**			
PGY-1	21 (33.9%)	22 (35.5%)	0.92
PGY-2	24 (38.7%)	23 (37.1%)	
PGY-3	17 (27.4%)	17 (27.4%)	
Prior breast oncology rotation, *n* (%)	19 (30.6%)	21 (33.9%)	0.69
Prior exposure to AI-based learning, *n* (%)	11 (17.7%)	9 (14.5%)	0.63

Continuous variables were compared using Student's t-test, and categorical variables were compared using the χ^2^ test or Fisher's exact test, as appropriate.

### 3.2 Knowledge acquisition and clinical competency development

**Figure 2:**
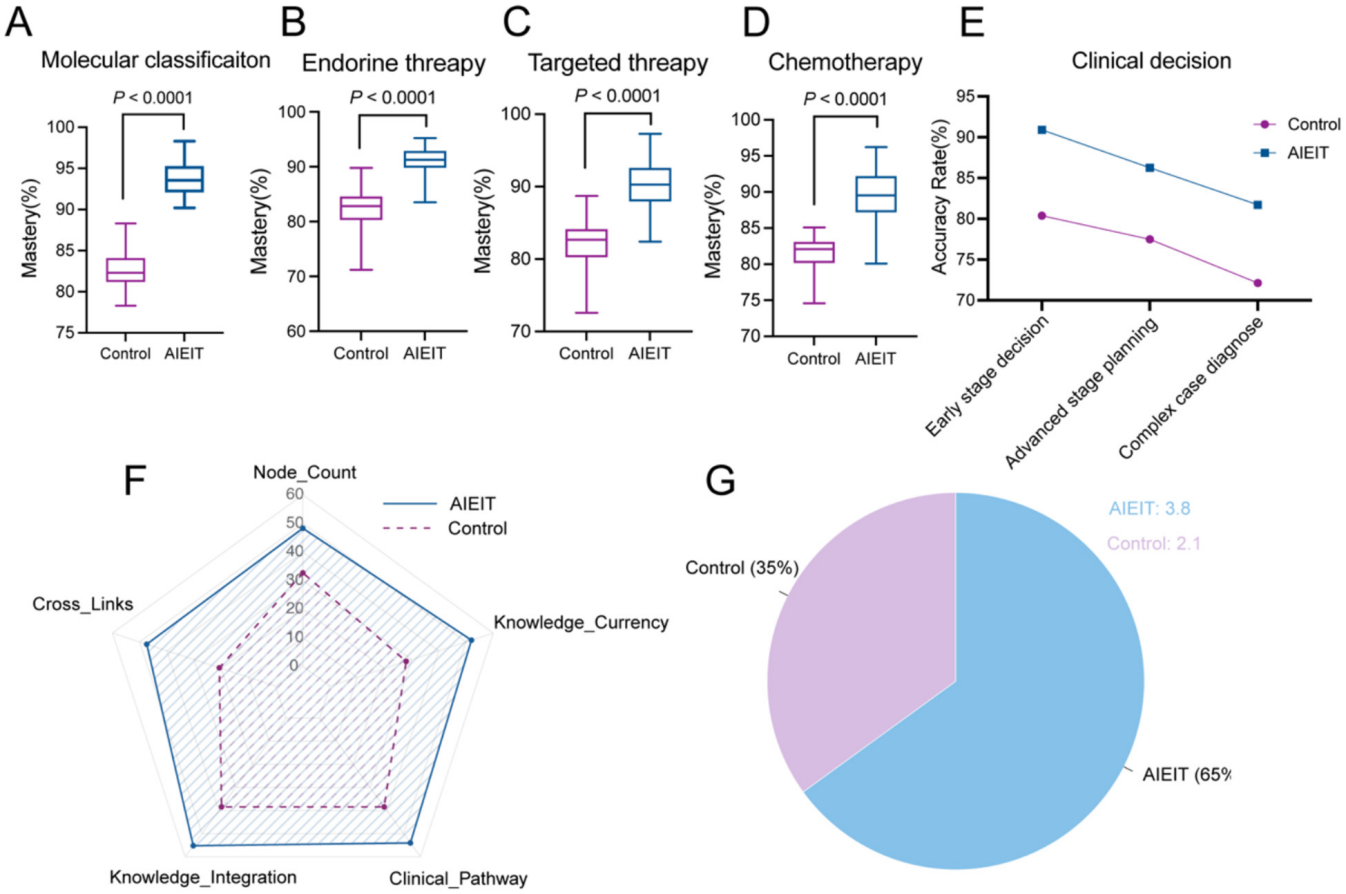
Comprehensive Evaluation of Core Competencies. **(A–D)** Box plots showing the accuracy of molecular classification and decision-making across four treatment domains—molecular classification **(A)**, endocrine therapy **(B)**, targeted therapy **(C)**, and chemotherapy **(D)**. **(E)** Comparison of clinical decision-making accuracy across task complexity levels. The AIEIT group consistently outperformed the control group in early-stage treatment, advanced-stage assessment, and complex case diagnosis. **(F)** Concept-map analysis illustrating differences in knowledge structure and reasoning organization between groups. **(G)** Evidence-based practice performance, showing the average number of evidence-based references cited per case.

The AIEIT group demonstrated significantly higher levels of specialized knowledge proficiency, defined as the percentage of correctly completed items within standardized, domain-specific assessments aligned with residency training objectives. Compared with the control group, the AIEIT group achieved higher proficiency in molecular classification (93.66 ± 1.92% vs. 82.65 ± 2.10%, *P* < 0.001; Figure 2A), endocrine therapy selection (90.94 ± 2.64% vs. 82.35 ± 3.50%, *P* < 0.001; Figure 2B), targeted therapy decision-making (90.07 ± 3.16% vs. 82.18 ± 3.83%, *P* < 0.001; Figure 2C), and chemotherapy strategy selection (89.39 ± 3.57% vs. 81.22 ± 2.36%, *P* < 0.001; Figure 2D). In clinical reasoning assessments, the AIEIT group demonstrated higher accuracy in early-stage (80.4%) and advanced breast cancer treatment decision-making (77.48%), as well as in the diagnosis of complex cases (72.13%), compared with the control group (*P* < 0.001 for all; Figure 2E). Concept-map analysis revealed a more integrated knowledge structure in the AIEIT group, with a greater number of nodes (48.3 ± 6.2) and a 2.3-fold increase in cross-links relative to the control group, indicating enhanced knowledge connectivity and synthesis (*P* < 0.001; Figure 2F). In evidence-based practice tasks, participants in the AIEIT group cited a higher mean number of high-level references per case than controls (3.8 vs. 2.1, *P* < 0.001; Figure 2G), reflecting greater utilization of evidence during clinical decision-making.

### 3.3 Technical skills and clinical performance

The AIEIT group demonstrated superior performance in technical skills and procedural assessments (Table 2). In imaging-based evaluations, breast ultrasound BI-RADS classification accuracy was higher in the AIEIT group than in the control group (88.9 ± 4.1% vs. 75.4 ± 6.2%, *P* < 0.001). Similarly, breast MRI interpretation accuracy was higher in the AIEIT group (85.7 ± 5.3% vs. 72.1 ± 7.5%, *P* < 0.001). In procedural assessments, the success rate of ultrasound-guided biopsy was higher in the AIEIT group (92.3% vs. 81.5%), with a shorter mean operation time (8.5 ± 2.1 min vs. 14.2 ± 3.8 min, *P* < 0.001). In surgical simulation tasks, the AIEIT group achieved higher margin prediction accuracy (89.4 ± 3.8% vs. 78.9 ± 5.6%, *P* < 0.001) and aesthetic outcome scores (86.7 ± 4.2 vs. 74.3 ± 6.1, *P* < 0.001). Skill retention assessed 3 months after training remained higher in the AIEIT group (91.2 ± 3.5% vs. 76.8 ± 8.4%, *P* < 0.001). Additionally, skill transfer to novel clinical scenarios was more frequently observed in the AIEIT group (83.7% vs. 68.5%, *P* < 0.001), suggesting improved procedural consolidation and adaptability.

**Table 2:** Comparative assessment of training outcomes.

**Assessment domain**	**Specific metric**	**Control**	**AIEIT**	***P*-value**
Specialized technical skills	Breast ultrasound BI-RADS classification accuracy (%)	75.4 ± 6.2	88.9 ± 4.1	<0.001
	Breast MRI evaluation accuracy (%)	72.1 ± 7.5	85.7 ± 5.3	<0.01
Procedural skills	Success rate of ultrasound-guided biopsy (%)	81.5	92.3	<0.05
	Operation time (minutes)	14.2 ± 3.8	8.5 ± 2.1	<0.001
Surgical simulation	Margin prediction accuracy (%)	78.9 ± 5.6	89.4 ± 3.8	<0.001
	Aesthetic assessment score (points)	74.3 ± 6.1	86.7 ± 4.2	<0.01
Skill consolidation & adaptability	Skill retention rate (3-month, %)	76.8 ± 8.4	91.2 ± 3.5	<0.001
	Skill transfer success rate (%)	68.5	83.7	<0.05

Continuous variables are presented as mean ± standard deviation and were compared between groups using Student's *t*-test. Categorical variables are presented as percentages and were compared using the χ^2^ test. A two-sided *P* value <0.05 was considered statistically significant.

### 3.4 Collaborative competence and professional literacy enhancement

Across collaborative and professional domains (Table 3), the AIEIT group consistently achieved higher performance scores than the control group. In core competency assessments, the AIEIT group scored higher in case presentation (93.4 ± 3.2 vs. 82.6 ± 4.8, *P* < 0.001), imaging interpretation (91.2 ± 3.8 vs. 79.4 ± 5.2, *P* < 0.001), and treatment planning (89.7 ± 4.1 vs. 78.1 ± 4.6, *P* < 0.001).Team collaboration assessments demonstrated higher communication efficiency (4.4 ± 0.3 vs. 3.6 ± 0.4, *P* < 0.001), a greater proportion of active listening behaviors (45.3% vs. 29.5%, *P* = 0.002), and more frequent spontaneous leadership behaviors (76.8% vs. 42.7%, *P* < 0.001) in the AIEIT group. Professional literacy scores were also higher in the AIEIT group for patient communication (4.5 vs. 3.9, *P* = 0.004) and ethical decision-making (4.6 vs. 4.0, *P* = 0.003). Burnout incidence was lower among participants in the AIEIT group compared with controls (35.2% vs. 61.5%, *P* < 0.001).

**Table 3:** Comparative analysis of professional competency, collaboration, and well-being.

**Assessment domain**	**Category**	**Control (Mean ±SD/%)**	**AIEIT (Mean ±SD/%)**	***P*-value**	**Interpretation**
Core competency performance (Points)	Case presentation	82.6 ± 4.8	93.4 ± 3.2	<0.001	Markedly improved case organization and clarity
	Imaging interpretation	79.4 ± 5.2	91.2 ± 3.8	<0.001	Higher diagnostic accuracy and reasoning
	Treatment planning	78.1 ± 4.6	89.7 ± 4.1	<0.001	Stronger evidence-based decision-making
Team collaboration assessment	Communication efficiency	3.6 ± 0.4	4.4 ± 0.3	<0.001	More effective and concise teamwork
	Active listening behaviors	29.5 %	45.3 %	0.002	Greater frequency of attentive interaction
	Natural leadership	42.7 %	76.8 %	<0.001	Majority assumed spontaneous coordinating roles
Professional literacy (5-point scale)	Patient communication	3.9	4.5	0.004	Enhanced empathy and patient-centered dialogue
	Ethical decision-making	4.0	4.6	0.003	Stronger moral reasoning and professionalism
Occupational Well-being	Burnout incidence	61.5 %	35.2%	<0.001	Significantly reduced emotional exhaustion

### 3.5 Learning process and cognitive efficiency analysis

**Figure 3:**
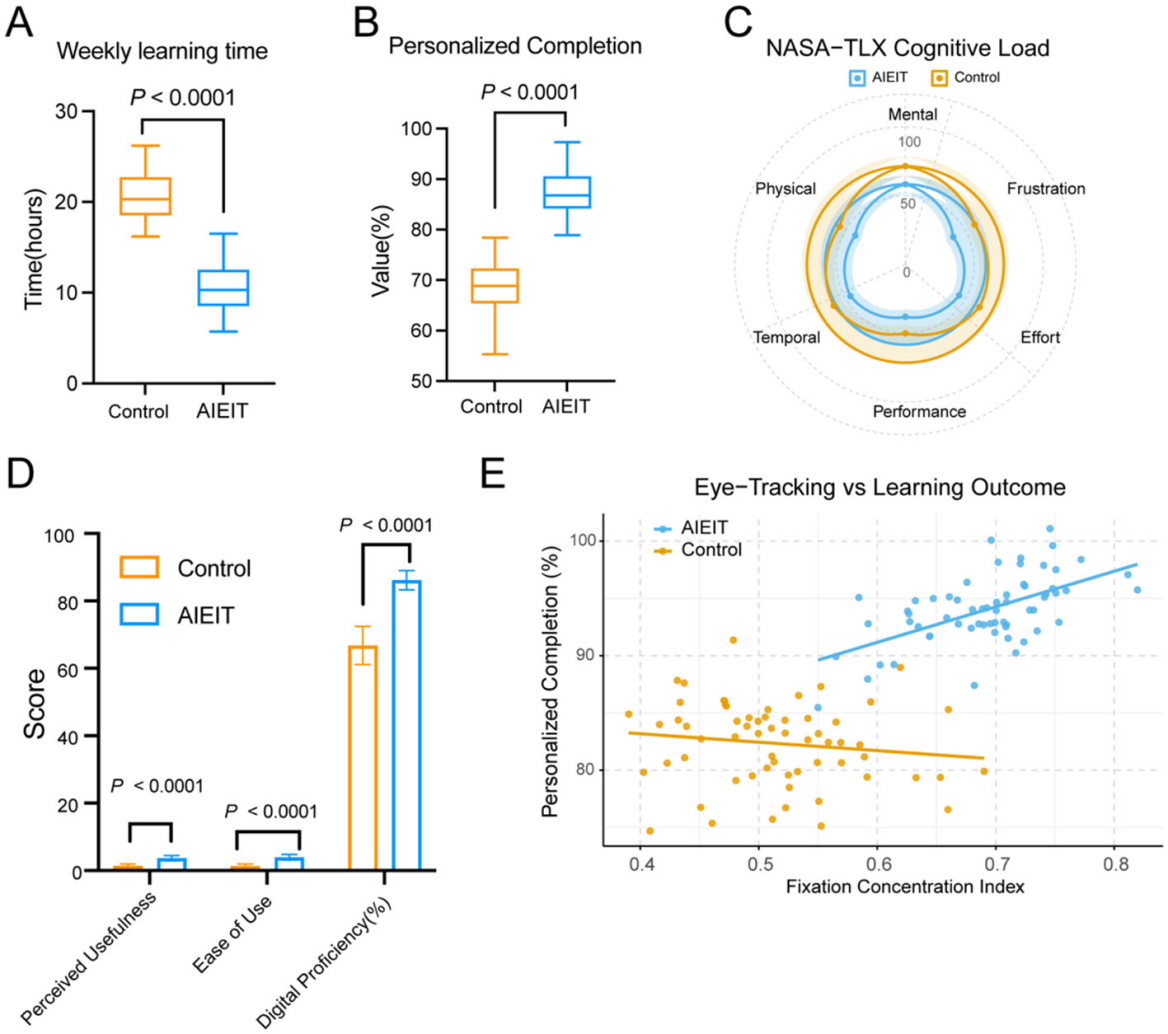
Learning analytics and cognitive efficiency assessment. **(A)** Weekly learning time. Box plot showing the distribution of average weekly study duration. **(B)** Personalized completion rate. Box plot showing the completion percentage of individualized learning content. **(C)** NASA–TLX cognitive load evaluation. Radar chart illustrating multidimensional cognitive load scores, including mental, physical, temporal, performance, effort, and frustration components. **(D)** Technology acceptance and platform proficiency. Bar graph displaying the mean scores for perceived usefulness, ease of use, and digital proficiency. **(E)** Eye-tracking and learning outcome analysis. Scatter plot showing the relationship between fixation concentration index and personalized completion percentage, with point size representing the improvement in key-information capture.

The AIEIT group exhibited higher learning efficiency and engagement. Weekly learning time was shorter (10.63 ± 2.54 h vs. 20.62 ± 2.48 h, *P* < 0.0001; Figure 3A), while personalized content completion rates were higher (87.43 ± 4.19% vs. 68.58 ± 5.80%, *P* < 0.0001; Figure 3B). Cognitive workload assessed using the NASA–TLX scale showed lower mental, physical, and temporal demands, accompanied by higher perceived performance and lower frustration in the AIEIT group (all *P* < 0.05; Figure 3C). Technology acceptance and digital proficiency were evaluated using the same validated questionnaires in both groups (Figure 3D). Participants in the AIEIT group completed the assessment with reference to the AI-empowered intelligent teaching platform, whereas control participants completed the questionnaire based on the conventional digital learning tools routinely used in standard residency training, enabling a standardized comparison of technology-related learning experiences. The AIEIT group reported higher perceived usefulness (3.69 ± 0.80 vs. 1.45 ± 0.53), ease of use (3.95 ± 0.84 vs. 1.37 ± 0.58), and digital proficiency (86.18 ± 2.86% vs. 66.81 ± 5.65%; all *P* < 0.0001). Eye-tracking analysis demonstrated higher fixation concentration (0.70 vs. 0.50, *P* < 0.001) and higher task completion rates (94% vs. 82%) in the AIEIT group, with a positive correlation between fixation concentration and task completion (*r* = 0.63, *P* < 0.001; Figure 3E).

### 3.6 Long-term effects and comprehensive system benefits

**Figure 4:**
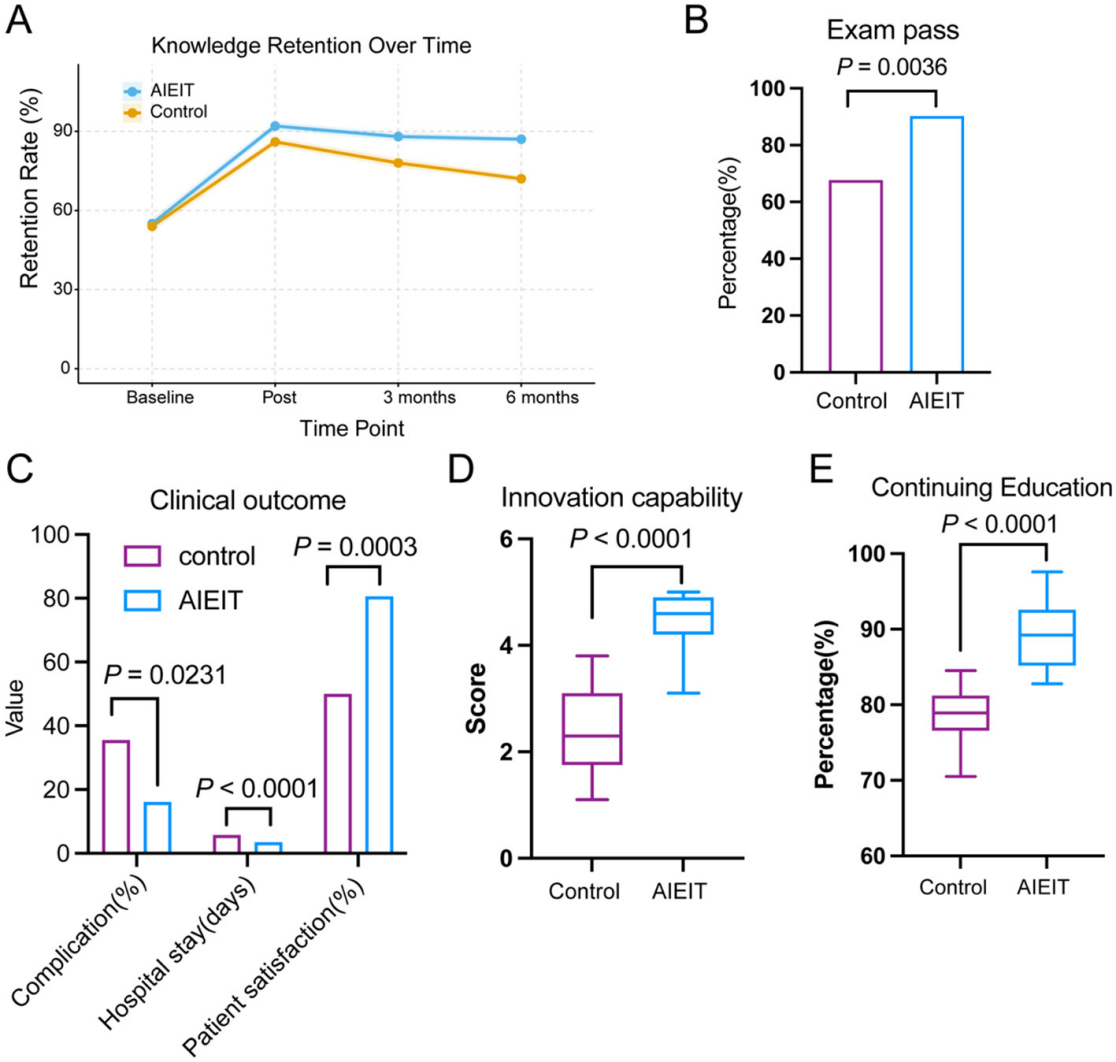
Long-term outcomes and professional development assessment. **(A)** Knowledge retention over time. Line graph showing knowledge retention rates measured at baseline, immediately post-training, and at 3- and 6-month follow-up time points. **(B)** Specialty examination performance. Bar chart presenting the percentage of participants achieving examination pass criteria. **(C)** Clinical practice outcomes. Bar chart displaying postoperative complication rate, average hospital stay duration, and patient satisfaction percentage. **(D)** Innovation capability. Box plot illustrating innovation capability scores assessed on a 5-point scale. **(E)** Continuing education participation. Box plot showing the percentage of participants engaged in continuing professional education activities.

At 6-month follow-up, the AIEIT group demonstrated higher knowledge retention compared with the control group (86.7 ± 4.2% vs. 74.2 ± 3.2%, *P* < 0.001; Figure 4A). Specialty examination pass rates were also higher in the AIEIT group (90.3% vs. 67.6%, *P* = 0.0036; Figure 4B). Patient-related clinical indicators were analyzed as exploratory outcomes. During the follow-up period, patients managed by residents in the AIEIT group were associated with lower postoperative complication rates, shorter hospital stays, and higher patient satisfaction compared with those managed by control group residents (all *P* < 0.05; Figure 4C). In addition, the AIEIT group demonstrated higher innovation capability scores (4.51 ± 0.46 vs. 2.39 ± 0.75, *P* < 0.0001; Figure 4D) and greater participation in continuing education activities (89.23 ± 4.31% vs. 78.65 ± 3.19%, *P* < 0.0001; Figure 4E), suggesting sustained professional development beyond the training period.

## 4 Discussion

This study demonstrates that the AI-enhanced intelligent teaching (AIEIT) model significantly improves residency training outcomes in breast oncology. Compared with conventional training approaches, AIEIT achieved consistent gains in knowledge acquisition, clinical skill proficiency, and multidisciplinary collaboration, addressing persistent challenges in oncology education such as fragmented knowledge delivery, limited structured clinical reasoning practice, and insufficient support for multidisciplinary decision-making.

The observed advantages of AIEIT can be attributed to three synergistic mechanisms that distinguish it from traditional training models. First, the dynamic knowledge graph continuously integrates updated clinical guidelines and emerging evidence, overcoming the static and instructor-dependent nature of lecture-based teaching while enabling personalized learning pathways. Second, the AI mentor system provides immediate feedback and adaptive case-based simulations, allowing repeated practice of diagnostic and therapeutic reasoning in a risk-free environment that is often difficult to achieve during routine clinical rotations. Third, the learning analytics dashboard shifts assessment from episodic summative evaluation to continuous, data-driven monitoring, enabling timely feedback and targeted remediation. Together, these components form an adaptive educational ecosystem that contrasts with conventional fixed curricula, opportunistic clinical exposure, and delayed feedback.

Compared with previously reported technology-assisted or simulation-based training models, AIEIT demonstrated particularly strong improvements in specialized oncology reasoning and multidisciplinary collaboration. The enhanced performance in virtual MDT simulations suggests that mixed-reality environments may better approximate authentic team-based clinical decision-making than traditional case discussions or single-discipline simulations, extending existing evidence on immersive educational strategies in complex clinical settings.

Notably, the AIEIT framework is inherently multimodal, integrating AI-driven personalization, mixed-reality simulation, and continuous learning analytics. Therefore, the observed effects cannot be attributed solely to AI components but rather reflect the combined influence of multiple interacting educational modalities. While this multimodality reflects real-world educational innovation, it introduces potential confounding when isolating the contribution of individual components. Future studies using factorial or component-level designs are needed to clarify the relative roles of AI, mixed-reality simulation, and analytics-driven feedback.

Beyond educational effectiveness, the AIEIT model demonstrated favorable indicators of sustainability, including more efficient use of instructor time and reduced reliance on physical training resources. The stability of skill acquisition and superior long-term knowledge retention suggest that the model promotes deep, integrative learning rather than short-term memorization.

Several limitations should be acknowledged. This study was conducted in urban tertiary teaching hospitals, potentially limiting generalizability to resource-limited settings. Randomization was not stratified by center, and residual center-level effects cannot be excluded. Multiple outcomes were assessed, increasing the risk of type I error despite hierarchical analysis. Some assessments were partially subjective, and patient-related outcomes were exploratory and should be interpreted cautiously. Longer follow-up is required to assess sustained career-level impact, and a Hawthorne effect related to novel technology exposure cannot be entirely excluded.

Future research should evaluate the scalability of the AIEIT framework across diverse specialties and institutional contexts. Incorporating generative AI for adaptive case generation and conducting long-term multicenter follow-up studies will be essential to further define the educational and clinical impact of intelligent teaching systems.

## 5 Conclusion

In conclusion, the AIEIT model represents a transformative advancement in medical education. Through data-driven personalization, human–AI collaboration, and virtual–real integration, it provides a scalable, sustainable framework for cultivating competent, adaptive, and future-ready oncologists in the era of digital intelligence.

## Data Availability

The datasets presented in this study can be found in online repositories. The names of the repository/repositories and accession number(s) can be found in the article/supplementary material.
